# Estimation and Evaluation of Future Demand and Supply of Healthcare Services Based on a Patient Access Area Model

**DOI:** 10.3390/ijerph14111367

**Published:** 2017-11-10

**Authors:** Shunsuke Doi, Hiroo Ide, Koichi Takeuchi, Shinsuke Fujita, Katsuhiko Takabayashi

**Affiliations:** 1 Department of Healthcare and Information Management, University of Tokyo Hospital, 7-3-1 Hongo, Bunkyo-ku, Tokyo 113-8655, Japan; 2 Department of Welfare and Medical Intelligence, Chiba University Hospital, 1-8-1 Inohana, Chuo-ku, Chiba 260-8677, Japan; ide@chiba-u.jp (H.I.); koichist@chiba-u.jp (K.T.); fujitan@faculty.chiba-u.jp (S.F.); 3 Department of Internal Medicine, Sanwa Hospital, Medical Incorporated Association Kanae-kai, 7-379 Higurashi, Matsudo, Chiba 270-2253, Japan; takaba@kanae-kai.or.jp

**Keywords:** geographic information systems, health services demand, health services geographic accessibility, estimation

## Abstract

Accessibility to healthcare service providers, the quantity, and the quality of them are important for national health. In this study, we focused on geographic accessibility to estimate and evaluate future demand and supply of healthcare services. We constructed a simulation model called the patient access area model (PAAM), which simulates patients’ access time to healthcare service institutions using a geographic information system (GIS). Using this model, to evaluate the balance of future healthcare services demand and supply in small areas, we estimated the number of inpatients every five years in each area and compared it with the number of hospital beds within a one-hour drive from each area. In an experiment with the Tokyo metropolitan area as a target area, when we assumed hospital bed availability to be 80%, it was predicted that over 78,000 inpatients would not receive inpatient care in 2030. However, this number would decrease if we lowered the rate of inpatient care by 10% and the average length of the hospital stay. Using this model, recommendations can be made regarding what action should be undertaken and by when to prevent a dramatic increase in healthcare demand. This method can help plan the geographical resource allocation in healthcare services for healthcare policy.

## 1. Background and Purpose

Along with improvement in medical environments and hygiene, Japan has become a country with one of the longest lifespans. However, the rapid improvement in the mortality rate has combined with the decline in birthrate to cause a major skew in the composition of the population, resulting in Japan having the largest population ratio of persons aged 65 years or older in the world [1]. Furthermore, Japan is expected to become the most rapidly aging society in history, with the population aged 65 years or older in the Tokyo metropolitan area increasing by 27% in only 10 years from 2010 (National Institute of Population and Social Security Research [2]). This is because the large population group known as the “baby boomer generation”, who were born between 1947 and 1949 (following the Second World War), will all become elderly at once. The largest problem is the concentration of elderly persons (over 10 million persons) in the near future in the Tokyo metropolitan area, a small area with high land and commodity prices. Considering the human and economic resources related to care for the elderly, such as medical care and nursing care services, it is easy to imagine the magnitude of the social burden this will cause.

As the number of elderly persons increases, it is predicted that the healthcare services supply capacity will eventually fall significantly short of the demand [3]. To prepare for such a situation, it is necessary to accurately estimate future healthcare demands. However, unlike the healthcare systems in Western countries, Japan has a “free access system”, in addition to the comprehensive healthcare insurance system, which allows patients to freely visit any medical institution without referral [4]. There is no gate keeper system as the general practitioner (GP) system. Clinic doctors are in charge of primary care. In hospital care, secondary healthcare areas (SHAs) have been established to help manage total healthcare services supply. In Japan, a healthcare plan, which includes the management of the number of hospital beds, is formulated in each SHA. For example, Tokyo has 13 SHAs and 104,140 beds, which is described in the Tokyo Metropolitan Healthcare Plan 2015. However, because there is no restriction on the health-seeking behaviours of residents, a few of the residents visit a hospital outside of the SHA in which they live. Under this system, it is not easy to accurately predict the number of patients per area and per medical institution (i.e., healthcare demand). Furthermore, from the standpoint of the medical providers (e.g., in Japan, Ministry of Health, Labour, and Welfare), examining how patients access medical treatment is to consider how to equally provide healthcare services. Nonetheless, far less emphasis has been placed on accessibility when a patient visits a medical institution in the Tokyo metropolitan area. Although the population density of the Tokyo metropolitan area is very high, because there are many medical institutions, most residents have access to an institution in the immediate vicinity of their residence. However, population aging modifies the spatial distribution of healthcare services demand. The government is afraid of the dramatic increase of healthcare services demand in the Tokyo Metropolitan Area.

Accessibility to healthcare services has a variety of interpretations of the concept, such as geographic access, cost of care, health literacy, and so on. Lesvesque et al. conceptualized accessibility in five dimensions: (1) approachability; (2) acceptability; (3) availability and accommodation; (4) affordability; and (5) appropriateness [5]. In this paper, we focus on the geographic accessibility of residents to healthcare service institutions, which corresponds to the concept of “availability” according to the Lesvesque’s classification.

Geographic information systems (GIS) have been used in recent years to analyse the geographic accessibility of residents to healthcare service institutions [6,7,8]. GIS is a powerful tool to investigate geographic accessibility between residents and healthcare service institutions. Therefore, GIS is used to analyse problems related to spatial distribution, such as healthcare service supply in rural areas, which are inconvenient in terms of public transportation [9], and the allocation of emergency care institutions [10]. In this paper, we devised a simulation model that applies this accessibility information to the estimation of healthcare supply and demand. This model is called the patient access area model (PAAM). The PAAM is a simulation technology that estimates the balance between supply and demand of healthcare services in the future based on Monte Carlo sampling [11] employing GIS technology. For example, it is necessary to consider how to secure accessibility for residents in areas where there is increased demand that cannot be dealt with by existing healthcare resources. To know when and where the demand exceeds the capacity of healthcare services is important. These are inevitable problems to address in light of the future system of healthcare supply, and estimation can be realized by utilizing the PAAM.

In this paper, we shall introduce an example of the application of the PAAM for estimating the future balance of supply and demand of healthcare in the Tokyo metropolitan area while taking accessibility into consideration.

## 2. Materials and Methods

There are three preconditions for using the method introduced in this section. First, the reference population for estimating demand must be aggregated from a set of small areas. In Japan, there is the national census including population data in each small area, and these data have been published on the government website. For example, the Japanese Statistics Bureau of the Ministry of Internal Affairs and Communications publishes data concerning population by sex and age group as electronic data for each standard area mesh of the World Geodetic System [12]. Second, network information is needed to perform access analysis by GIS. Network information comprises the route and distance to travel on a map (e.g., a roadmap or railroad map), mean time needed to travel, and is essential for calculating the time required for actual travel by means such as walking or automobile. Many developed countries, including Western countries, have such network information available. Third, the minimum amount of statistical data necessary for simulation, such as population dynamics (e.g., birth rate, mortality rate) and actual number of patients, must be prepared as parameters for estimating demand (e.g., future population and number of patients).

### 2.1. Approach of the PAAM

This section provides an overview of the PAAM and a summary of its calculation methods. The PAAM is a method for estimating future medical demand while ensuring the accessibility of patients to medical institutions (time distance). Guarantee of access is realized by limiting patients needing treatment at a hospital to a certain geographic range based on the time distance. As a result, from the perspective of patients, it becomes possible to visit a healthcare services institution within a certain period of time. This time distance is set for all healthcare service institutions based on a traffic analysis utilizing GIS. Since it is not possible to know where a patient will arise in the future, we created an algorithm that randomizes the occurrence of patients within each district based on Monte Carlo sampling, in order to estimate the future population and number of patients in each area.

In the present study, we used the PAAM to model the hospital access of patients in each small area in order to estimate demand. The overall flow included the following four steps:

(1) Estimate the future population of each small area and the number of future patients;
(2) Set the range in which patients can receive healthcare services (setting patient access area);
(3) Simulate patients’ selection of a healthcare services institution (modelling health-seeking behaviours); and
(4) Determine small areas where there is over-demand (evaluation of the balance between supply and demand).

In the approach of the PAAM, we defined the demand as residents who need healthcare services (e.g., the number of patients) and the supply as the capacity of healthcare service institutions (e.g., the number of hospital beds).

In Japan, as previously mentioned, the census population for every 1/2 standard area mesh (500-m mesh) and the ratio of patients per unit population for each prefecture/sex/age group are available to the public. Therefore, in the present study, we defined “small areas” as 500-m meshes.

#### 2.1.1. Estimation of Future Populations of Small Areas and Number of Future Patients

First, we estimated the future population of each mesh using the current population composition and certain statistical indices. As a method of estimating the future population, we used the primary factors cohort method, which is commonly used in the field of population estimation. The parameters used here are the population of each small area according to gender and age group, as well as the birth rate, male-to-female birth ratio, mortality rate, net migration rate, etc., of the area. In Japan, the Ministry of Health, Labour, and Welfare and the National Institute of Population and Social Security Research publish these statistics for each municipality [13]. In a primary factors cohort, the reference population is multiplied by these parameters to obtain the expected future population. However, in small areas, there are cases where the population size is exceedingly small—in fact, depending on the age group, there would be many areas where the expected population is less than one person. Moreover, because population migration and death occur in a stochastic manner, stochastic demographics are required when performing a simulation. Therefore, for population estimation in small areas, we used the Monte-Carlo sampling to stochastically provide parameter values while always sampling births, movement, and deaths in the population on a per person basis. As a result, the estimated population for each small area fluctuates with each sampling.

Next, we estimated the number of future inpatients (hospitalized patient) from the estimated population. The parameters used for calculating the number of inpatients per day, such as the average length of hospital stay (average number of days of inpatient hospital stay) and rate of inpatient care (number of people in the population receiving inpatient treatment per day). The Ministry of Health, Labour, and Welfare publishes these statistics by prefecture. These values represent the incidence of inpatients per day per population by sex, age, and disease classification. We estimated the total number of future inpatients in each area by multiplying the relevant statistical value by the aforementioned estimated future population. As for the future population estimation, the number of inpatients was stochastically calculated for each person using Monte Carlo sampling.

Additionally, it is possible to vary these parameters based on the assumptions of the estimation. For example, it has been indicated that the rate of inpatient care at a hospital in Japan has declined at a nigh-constant rate since 1990 [14]; thus, in the experiment in the present study, the future fluctuation of the rate of inpatient care at a hospital was included as a parameter.

#### 2.1.2. Setting of Patient Access Areas (PAAs)

**Figure 1:**
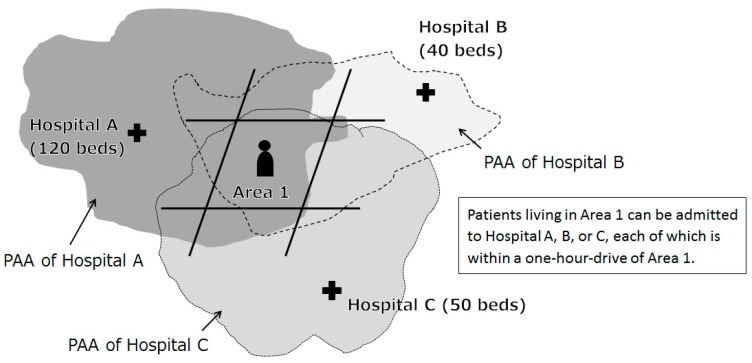
Approach of the PAAM.

In this section, we explain our method of setting PAAs, which focuses on patient time distance to medical facilities in order to ensure patient accessibility. As described in introduction, the area of health-seeking behaviours of residents do not always correspond to regional division. The PAAM models the health-seeking behaviours of patients by restricting institutions in which patients receive healthcare services in their PAA. Residents can choose any hospital within PAA and never mind the regional division. The PAA is a range in which travel to a certain healthcare service institution can be made within a certain time using a specific means of transportation. The approach of this model is shown in Figure 1.

From the point of view of the medical facilities, it means that the patient is hospitalized only from meshes within their own PAA. Similarly, from the viewpoint of patients, this means that a patient can visit a given medical institution only if they live in the mesh that is within the PAA of that institution. Setting the PAA is easily the most important definition in the PAAM for simulating health-seeking behaviours.

To set the PAA, we performed a transportation analysis using network information, such as roads and railroads in the area, as well as GIS software. The PAA essentially calculates the time distance from each mesh to each medical institution for various means of transportation, such as walking, bicycle, automobile, or railway. The upper limits of the means of transportation and time distance can be set as parameters, and may be standardized for all institutions or be allowed to vary according to the size and role of the institution. Figure 1 shows a case in which the time is uniformly set to 1 h by car.

#### 2.1.3. Modelling Patients’ Health-Seeking Behaviours

By setting a PAA, healthcare service institutions (i.e., hospital) within a set time distance from each small area can be visualized. The problem here is that each mesh does not necessarily correspond to a single PAA; often, the PAAs of multiple healthcare service institutions will overlap in a given area. In such cases, it is necessary to simulate patients’ selection of healthcare service institutions in their mesh. This selection is referred to as “allocation” in the PAAM.

In the example in Figure 1, a patient in Area 1 can be allocated to hospital A, B, or C. However, each facility can only accept a limited number of patients, which depends on their supply capacity. In the field of hospitalized medicine, supply capacity is reflected by bed number. In the example in Figure 1, the supply capacity of the three facilities is 210 beds (the sum of the number of beds of the three facilities = 210). In addition, how many beds are used per day differs for each medical institution, depending on the function of the institution. Therefore, “hospital bed utilization rate”, which is the rate of use of the beds, was incorporated as a parameter. For example, if the hospital bed utilization rate is 80% for the aforementioned 210 beds, the number of available hospital beds is only 168 beds. The hospital bed utilization rate in Japan, depending on the function of the hospital, ranges from approximately 75% to 95%, in general [15].

Additionally, there are multiple methods of allocation, depending on the estimation policy (e.g., prioritizing large-scale facilities or time and distance to facilities, or stochastically distributing patients to each facility). Depending on the distribution method of patients, the number of patients that can be hospitalized varies greatly. In general, the Huff model is used, which is proportional to the attractiveness degree (such as the sales floor area) of the facility and inversely proportional to the distance to the facility. In the present study, we propose Monte Carlo sampling as a distribution method based on the health-seeking behaviours of patients, whereby the residents themselves stochastically select hospitals. Details will be explained in the next section.

#### 2.1.4. Evaluation of Balance between Supply and Demand and Prediction of Areas with Excess Healthcare Demand

The number of healthcare service institutions and the number of beds in the target area are limited. Additionally, the location of medical institutions is not uniform, but rather is mainly skewed towards areas with concentrated populations. Therefore, it can be expected that some medical institutions are lacking in supply capacity (i.e., the number of beds), while other institutions have a surplus of beds.

In the present study, the estimated number of inpatients within the target area was defined as the total demand, and the number of patients equal to the number of available hospital beds (derived by multiplying the number of beds in each medical institution by the hospital bed utilization rate) was defined as the upper limit of the total supply quantity. Furthermore, by simulating the health-seeking behaviours of patients, we were able to compare the estimated future supply and demand for each year. As for the method of evaluating the balance between supply and demand, we defined a state in which a patient cannot be hospitalized within a PAA as “over-demand”. By contrast, the state wherein the number of hospital beds of the medical institution is excessive (i.e., there is a surplus of vacant beds) is defined as “over-supply”. This enables us to evaluate “over-demand” for each mesh and “over-supply” for each medical institution. Furthermore, by mapping meshes with such over-demand, it is possible to visually indicate in which parts of the target area excess demand will occur. Then, by creating one for each year to be estimated, we can predict the changes over time.

### 2.2. Evaluation Experiment Regarding the Balance between Healthcare Supply and Demand in the Tokyo Metropolitan Area

In the present study, we applied the PAAM and conducted a simulation experiment, using the Tokyo metropolitan area as the target area. In this section, we shall explain the assumptions and parameters used in this experiment for each process of the PAAM.

#### 2.2.1. Target Areas and Population

The target area comprises the Tokyo metropolitan area and three adjacent prefectures; the total population was approximately 35 million persons in 2010, and the area is 13,557 km^2^. In the target field, there were 1623 hospitals with 258,794 beds. The estimation period was set as every five years from 2010 to 2040, and the 2010 population was based on the national census population. The small area unit was defined as one-half the size of the standard area mesh in the World Geodetic System. As the Earth is spherical, this mesh is not an exact square, but it has a shape approximate to a square that is 500 m in length on one side.

Population by sex/five-year age group in the 2010 census data was used as the data for estimating the standard population. However, for meshes that are less populated and in which there is a risk that personal information may be identified, we added the population to the surrounding meshes. Population groups with unknown sex and age were proportionally allocated in advance based on the age class composition distribution of each mesh.

#### 2.2.2. Parameter Setting in Estimating Future Population and Future Number of Patients in Each Small Area

Data were obtained from the following sources to define the parameters for estimating the future population: the birth rate and male-to-female birth ratio by prefecture were based on the 2011 Population Dynamics Survey of the Ministry of Health, Labour, and Welfare, while the March 2013 estimated survival rate and net migration rate by the local municipality were calculated by the National Institute of Population and Social Security Research. The rate of inpatient care (by sex, five-year age group, general classification of disease, and prefecture), which was used to calculate the future number of patients, was obtained from the 2011 Population Dynamics Survey of the Ministry of Health, Labour, and Welfare.

However, these are current values, and are unlikely to remain constant in the future. In fact, due to increasing healthy life expectancy and greater efficiency of medical treatment, the rate of inpatient care has continually declined. As such, it is necessary to set parameters with consideration of future fluctuations when estimating the number of future patients. However, changes in the healthcare provision system and society cannot be represented by quantitative changes, and these changes are not unidirectional. Furthermore, factors, such as rate of inpatient care and age composition, differ by area, making it difficult to accurately display these changes. Therefore, we account for changes in the estimation parameters and observed how these changes led to changes in the estimation results. Parameters incorporated into the estimation of the future number of inpatients were as follows:

(1) Changes in the rate of persons receiving inpatient treatment at a hospital due to disease control (rate of inpatient care); and
(2) Reduction in the average number of days of inpatient hospital stay due to improved healthcare efficiency (average length of hospital stay).

In the case of average length of hospital stay, it is generally not used for estimating the number of patients. However, as the number of days that a patient occupies a hospital bed decreases with the average length of hospital stay, the apparent number of patients per day per medical institution will also decrease. Therefore, in the present experiment, we decided to incorporate the reduction rate of the average length of hospital stay compared with the current figures as a parameter for estimating the number of inpatients.

Equation (1) shows the formula for estimating the number of future inpatients that considers these changes in parameters:

(1) $$N_{y}=\sum \left(P_{y,g,a}\times R_{y,g,a}\times \left(1-R_{\mathrm{ad}}\right)\times \left(1-D_{\mathrm{ad}}\right)\right)$$

where N is the number of patients, P the population, and R rate of inpatient care; they are shown as numerical values by estimation year (y), sex (g), and age class (a), respectively. Furthermore, R_ad_ is the rate of decrease of rate of inpatient care, and D_ad_ is the rate of reduction of the average length of the hospital stay.

#### 2.2.3. Parameter Settings for PAAs

To set the PAAs, each medical institution within a travel area of 60 min by car was identified using a GIS. This is based on our preliminary investigation, which showed that more than 70% of patients in the area used a car as a means of transportation at the time of admission and more than 80% of the patients are hospitalized in a medical institution within one hour from home [16]. The 1-h travel area was set as the area by which it was invariably possible to visit several hospitals from anywhere in the Tokyo metropolitan area.

#### 2.2.4. Assumptions and Parameter Setting for Modelling the Health-Seeking Behaviours of Patients

As mentioned in the previous section, in the PAAM, it is necessary to set the distribution method of patients according to the estimation policy. In the present study, in order to more realistically examine the supply-demand balance of inpatient healthcare in the target area, we created a model incorporating the free access system in medical institutions in Japan and the trends of health-seeking behaviours of patients and used them for the simulation.

**Figure 2:**
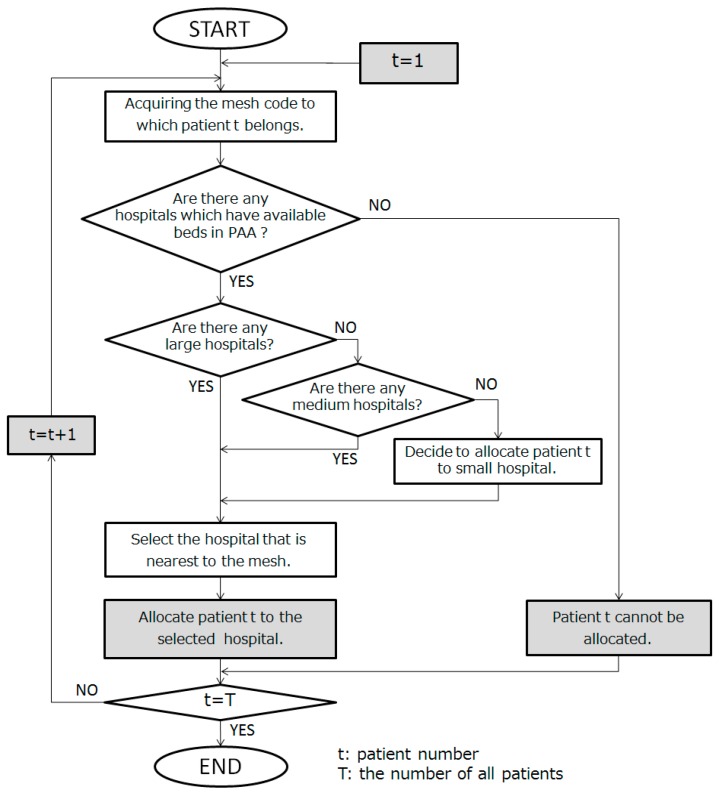
Simulation flow of selection of medical institution to be consulted by a patient.

The flow of the model is shown in Figure 2. All patients were prepared randomly in advance. Next, for each patient, hospitals were determined and allocated according to the hospital size and order of priority of time distance. This is because patients tend to go to large hospitals with well-equipped facilities and hospitals as close to home as possible. In accordance with previous studies, we defined hospital size as small scale (up to 100 beds), medium size (101–400 beds), or large scale (400 or more beds). We assumed that patients cannot be allocated when there is no bed in the PAA, and continued to repeat for all patients. By repeating this calculation, we investigated in which mesh undistributable patients appeared and whether there would be hospitals with empty beds.

Medical resources in the target area were obtained from the publicly available SHA database; the number of medical institutions with 20 or more hospital beds was set at 1629 and the number of hospital beds as 261,104. However, in reality, not all hospital beds are actually used. Thus, it is necessary to consider the utilization rate of hospital beds. This was based on the current mean value (80%) in medical institutions within the target area. Furthermore, because patients also move out of the region, of the medical institutions in the five prefectures adjacent to the target area that fell under the PAA, we allowed for the distribution patients in up to 20% of available beds.

#### 2.2.5. Evaluation of Balance between Supply and Demand and Scenario Setting in Prediction of Areas with Healthcare Over-Demand

As described in the previous section, in this experiment, a sensitivity analysis was performed by repeated experimentation with various assumptions and parameter settings. In the present paper, we explain three typical scenarios. The parameters to be incorporated in the simulation and the setting values of each scenario are shown in Table 1. In the simulations, sampling was performed 100 times for each scenario.

## 3. Results

### 3.1. Results of Estimation of Future Number of Inpatients

**Figure 3:**
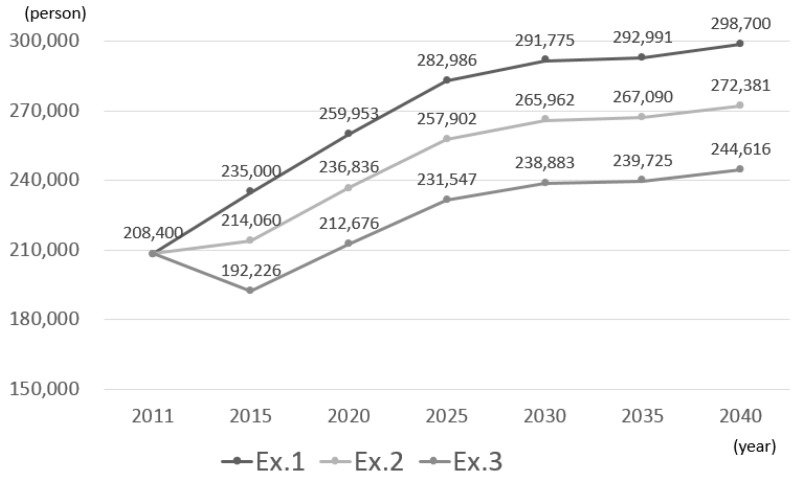
Estimated number of future patients.

**Figure 4:**
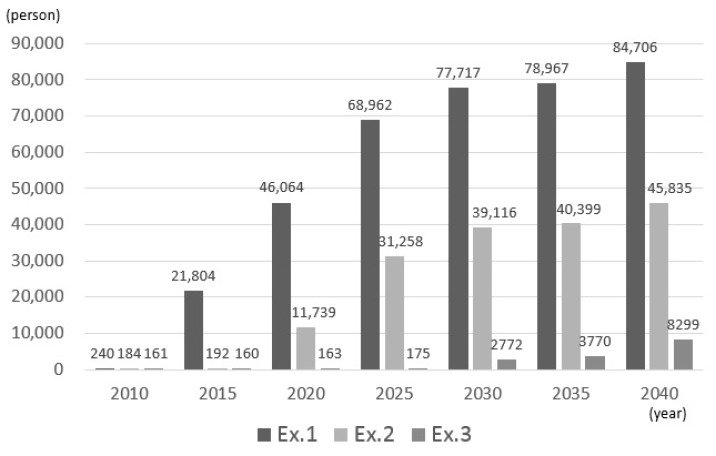
Estimated number of over-demand.

Figure 3 shows the estimated number of inpatients every five years. In the present experiment, because the estimated number of inpatients was estimated in a similar manner for each mesh similar as for the estimated population, the estimation results in Figure 4 were obtained by summing the estimated values of each mesh and taking the mean value of the sampling results.

The estimated number of inpatients was found to continue to increase with the population growth of elderly persons. Specifically, in experiment 1, it increases to approximately 300,000 persons per day in 2040. This is approximately 1.45 times the number in 2010. By contrast, the number of beds in the target area as of 2012 is only approximately 260,000 beds. Even when a simple comparison is made, assuming that the same healthcare continues, there will be a shortage of hospital beds by 2025. Compared with experiment 1, the number of patients decreased in experiments 2 and 3 due to the reduction in the average length of hospital stay and decrease in rate of inpatient care.

### 3.2. Evaluation Results of Supply–Demand Balance of Healthcare

The results of comparing the number of inpatients (amount of demand) and number of beds (amount of supply) in order to evaluate the healthcare supply–demand balance by using the PAAM are shown.

Figure 4 shows the number of patients who could not be hospitalized—that is, the number of patients who exceeded the demand—in each experiment. According to the estimation results, in the Tokyo metropolitan area, over-demand is expected as the number of patients increases. In experiments 1, 2, and 3, over-demand occurred from 2015, 2020, and 2030, respectively.

**Figure 5:**
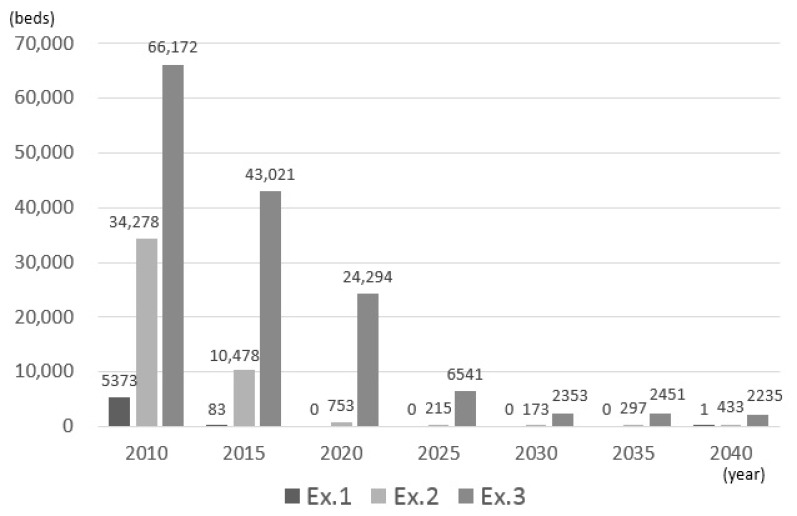
Estimated number of over-supply.

Figure 5 shows the number of hospital beds to which patients were not allocated—that is, the number of beds for which there was over-supply—in each experiment. The estimation results revealed that there were ample hospital beds in 2010, but the number decreases as the number of patients increases. In experiment 1, patients were allocated to almost all hospital beds starting from 2015. However, in experiment 2, even in cases where over-demand occurred, there was an over-supply depending on the area.

### 3.3. Results of Prediction of Areas with Excess Healthcare Demand

**Figure 6:**
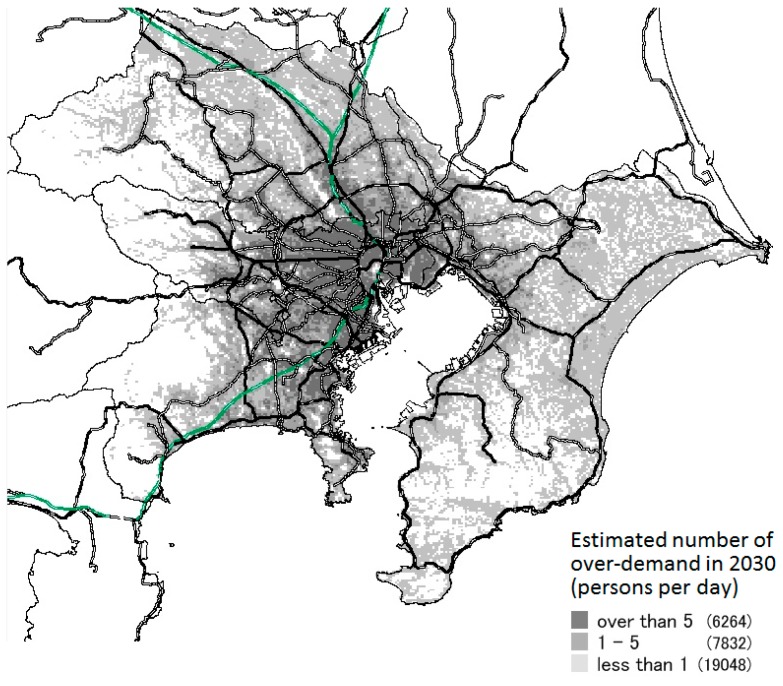
Estimated number of over-demand in each mesh (Ex.1).

**Figure 7:**
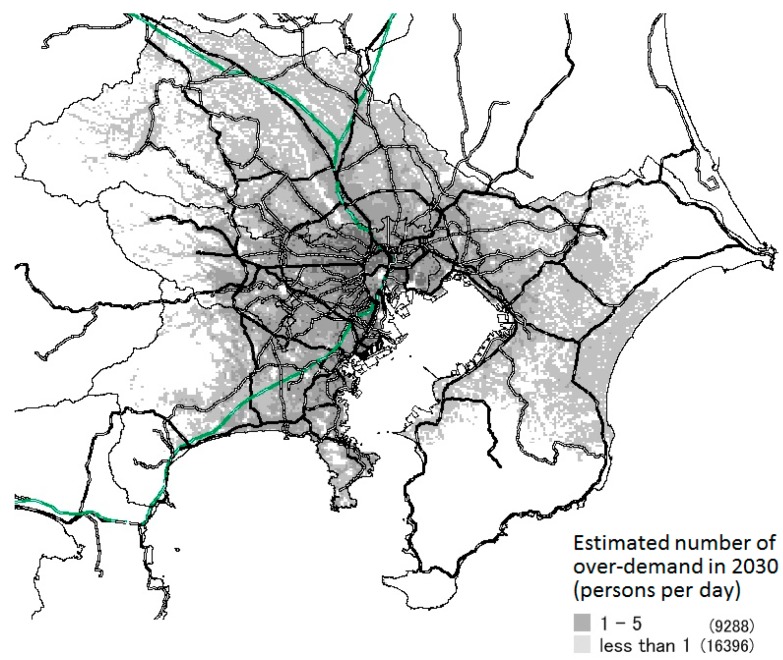
Estimated number of over-demand in each mesh (Ex.2).

**Figure 8:**
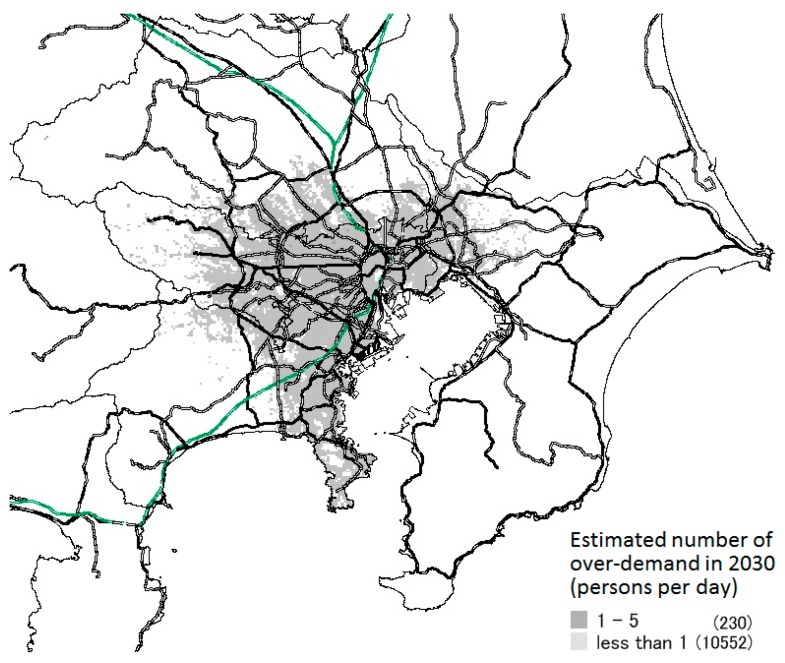
Estimated number of over-demand in each mesh (Ex.3).

Figure 6, Figure 7 and Figure 8 show the meshes in which over-demand occurs in 2030 in each scenario. The amount of over-demand is mapped for each mesh in Figure 6; the number of patients not allocated to hospital beds per day is shown. The bold lines on the map indicate the main lines of the Japan Railways/private railways.

Looking at this map, in experiment 1, a large over-demand is expected to occur mainly in the main areas of Tokyo, but in experiment 3, the over-demand was greatly relaxed. Moreover, over-demand will not occur in all areas of these prefectures or SHAs. Instead, the majority will occur in the vicinity of major transportation routes, such as national highways and railroads, and in areas with concentrated populations.

In experiments 1–3, when the total amount of over-demand in the area decreases, the number of meshes in which excess demand occurs decreases. Furthermore, the excess amount per one mesh also decreases. Moreover, although the total amount of over-demand differs for each scenario, the places in which the excess was concentrated were almost the same.

## 4. Discussion

The simulation results by the PAAM revealed that the number of inpatients in the Tokyo metropolitan area will sharply increase until 2030, and that there is a high possibility that areas with a shortage of hospital beds will occur mainly within the central Tokyo area. A future shortage was expected in all three experiments, suggesting that it is necessary to drastically reform the healthcare provision system in the future. Moreover, in mapping the areas with healthcare over-demand, we found that the areas with excess appear not over the entire area, but rather along railroads, national expressways, etc.

According to the government survey, patients prefer near and large-scale hospitals [17]. In the present study, by utilizing the PAAM, we made estimations under the assumption that hospitalization will be based on the inpatients’ health-seeking behaviours. Under this assumption, both the future over-demand and over-supply will occur as shown in the result of 2030 in Ex.3. This suggests if patients are hospitalized in a disorderly manner, areas where the number of hospital beds falls short of the demand will appear earlier. To solve this problem, it is better to plan the policy for the demand-side than to plan for the supply-side because it is not realistic to rely solely on the quantitative investment of medical resources in response to ever-increasing medical demand from the perspective of current finances and ensuring ample human resources. In fact, the average length of hospital stays in Japan is the longest in the Organisation for Economic Co-operation and Development (OECD) countries [18]. Instead, it is necessary to improve the efficiency of healthcare through a reduction in the length of hospital stays, introduction of critical paths, etc. However, as can be seen from the results of experiment 1 to experiment 3, measures only from the healthcare supply-side may not be able to counter the explosive increase in demand due to the aging population. As a countermeasure on the demand-side, it is important to advance policies on health education and disease prevention together.

The PAAM is an unprecedented estimation method that combines geographical information and statistical information to estimate healthcare demands. Various other agencies have similarly engaged in the estimation of future demand based on population estimates [19,20,21]. However, previous studies focused on either single medical institutions or administrative districts as estimation units. Thus, the present study has an advantage in that it provides estimates for an entire area in detailed units. By performing mapping using 500-m meshes as the unit of estimation, we predicted which areas would likely have over-demand (e.g., railroads, national roads). If we can accurately estimate the areas where future over-supply will occur, it will be possible to guide patients to other healthcare service institutions. Furthermore, by examining the trends, it is possible to predict how and when these countermeasures will be needed. In the previous healthcare plan, it was common to seek the total amount of supply in a large district like SHAs. Approach from the total amount of supply is of course necessary, but actual demand and supply excess or deficiency often occurs in very limited narrow area units as shown in the results of experiments. The PAAM helps policy-makers of local governments plan more realistically according to the situation of each area.

Problems concerning healthcare demand are closely related to problems related to population concentration in urban areas and population aging. These problems have been seen in other developed countries, which are likely to progress in a similar manner as Japan [22]. The present study focused on inpatient medical care in the Tokyo metropolitan area, which is one of the most populous areas in the world, but this method can be easily applied in countries where appropriate data infrastructure is in place, as the method is highly versatile. However, it seems that it is unnecessary to consider accessibility in countries where the health management of residents is relegated to a certain administrative district, as in the GP system in the United Kingdom. Still, it will be useful for countries that have directly introduced a free access system similar to that in Japan, or in developing countries that do not yet have a full-fledged healthcare system. Even in countries that have adopted a system similar to the GP system, accessibility must be considered for certain services (e.g., specialized medical institutions for diseases such as stroke and heart disease or services that go beyond standard healthcare such as nursing care and hospice care [6,23,24]). Furthermore, by calculating the potential demand in consideration of accessibility using our method, it is also possible to verify whether the actual GP zones are consistent with the potential demand.

The limitations of the present study are as follows. First, according to the Lesvesque’s classification, healthcare access has several concepts from the different points of view. In the present study, we focused on the “geographic accessibility”, that means only “availability”. Second, using the PAAM, inpatients were allocated in terms of patient geographic accessibility. However, in many cases of actual health-seeking behaviours of patients, the decision of the hospital in which patients are hospitalized depends on the functionality of the hospital or the medical institution initially visited. Therefore, it would be necessary to evaluate the method using historical data and other fields in order to construct a model to better fit the current situation. Third, when setting the PAAs of hospitals, time distance was set according to uniform criteria, but it would be necessary to tailor these parameters to the specific area. Fourth, treatment phases, such as type of disease and acute/recovery period, were not included in the estimations in this study. It is, therefore, necessary to investigate medical departments, types of hospital beds, average length of hospital stay, etc., for each hospital, which would require an enormous amount of information.

## 5. Conclusions

Using the PAAM, the model that focuses on patient accessibility using GIS, we developed and examined a method to evaluate the balance of future supply and demand of healthcare. Our results show that by mapping areas where there is over-demand for each 500-m mesh, we can estimate future supply and demand more precisely in comparison with conventional estimation methods. The application of this method can help formulate healthcare policy in the current aging society by providing suggestions about the necessary measures for areas exhibiting high healthcare demand, and the appropriate timing for implementing such measures.

## Figures and Tables

**Table 1:** Scenario and parameter setting value of each experiment.

Experiment No.	Scenario Name	Rate of Inpatient Care	Average Length of Hospital Stays	Hospital Bed Utilization Rate
Ex.1	Current projection	±0%	±0%	80%
Ex.2	5% Improvement scenario	−5%	−5%	85%
Ex.3	10% Improvement scenario	−10%	−10%	90%

Regarding the rate of inpatient care and average length of hospital stay, the ratio of each cohort group (by prefecture/sex/age group of population) in comparison to current values are shown.
